# Nutritional and Hygienic Quality of Raw Milk in the Mid-Northern Region of Algeria: Correlations and Risk Factors

**DOI:** 10.1155/2014/131593

**Published:** 2014-10-14

**Authors:** Soumeya Adjlane-Kaouche, Rafik Benhacine, Faiçal Ghozlane, Abderrahmane Mati

**Affiliations:** ^1^Département de Biotechnologie, Université Saad Dahlab, Somaa, 9100 Blida, Algeria; ^2^Institut Technique d'Elevage (ITELV), 16111 Baba Ali, Alger, Algeria; ^3^Département des Productions Animales, Ecole Nationale Supérieure d'Agronomie (ENASA), 16200 El Harrach, Alger, Algeria; ^4^Laboratoire de Biochimie Analytique et Biotechnologies (LABAB), Université Mouloud Mammeri, 15000 Tizi Ouzou, Algeria

## Abstract

This paper aims to study the overall quality of raw milk in the mid-northern region of Algeria. The analysis results showed a decrease in the average temperature for the delivery of 1,54°C with *P<0.001*. However, no significant variation (*P>0.05*) was observed in almost all the physical and nutritional parameters studied (pH, fat content, and protein content) between M1 and M2. The average contamination by total mesophilic aerobic bacteria (TMAB), coliforms, yeasts, molds, and different pathogens in samples taken at M1 showed significant changes at M2. This was confirmed by the decrease of reduction time of methylene blue (RTMB), about 54%. The variation was described as follows: (*P>0.05*) for yeasts and (*P<0.05*) for molds in M1 and M2, (*P<0.05*) for TMAB in M1, and (*P<0.001*) for TC, FC, and TMAB in M2. The analysis for the detection of *Salmonella* spp. showed no contamination in all samples tested, while antibiotic residues were detected in 35% of milks delivered. In conclusion, several risk factors have been identified in this study, namely, the effect of the season and the distance between the farm and the dairy unit.

## 1. Introduction

In Algeria, the national production of raw milk is estimated at 3,14 billion liters/year [1], achieved at 73% by a dairy cattle herd coming from the various crosses with imported races mainly from Europe, including France, Austria, and Germany. Only a third of cattle milk production is valued on industrial sector. Approximately, 80% of the milk collected is valued on the private sector. Annual consumption has evolved to reach 140 liters/capita in 2012. Approximately 80% are imported. Being a product with high nutritional value, milk is associated with the history of mankind. Whether raw or processed, it is known for its support for the growth and multiplication of germs in many optimal conditions. Indeed, its conservation at the farm and during transport can cause serious problems. Milk quality is significantly influenced by the parameters of storage and transport. High temperature (+8°C) promotes the growth of lactic acid bacteria, where the milk acidification, especially if the temperature is associated with unfavorable conditions of transport [2]. High temperatures also promote the growth of pathogenic bacteria such as* Staphylococcus aureus*,* Salmonella*,* Listeria monocytogenes*,* Escherichia coli*, and Clostridia [3]. However, high levels of bacteria in raw milk can affect the quality and shelf life of milk even if it is pasteurized [4, 5]. The presence of antibiotic residues in milk can sometimes be a danger to the consumer, triggering in rare cases allergy problems [6] and toxic accidents [7, 8] or still favoring the emergence of resistant microflora, especially being a major cause of disruptions in fermentation and maturation of dairywide consumption. Many works have been done on the qualities of raw milk at different levels of the dairy sector in Algeria. Aggad et al. [9] evaluated the hygienic quality of milk in western Algeria on samples from the same dairy, at the reception, having undergone different treatments while Ghazi et al. [10] and Hakem et al. [11] have focused in their works on samples taken from the production site to assess their microbiological quality. To our knowledge, no previous study has been conducted on the evaluation of the overall quality of raw milk and its evolution during transport. It is in this context that this present study is inserted. It aims at the determination of nutritional and hygienic characteristics of milk mixture contained in collector's tank before departure and upon arrival at different dairy processing units. This is in order to establish correlations between some parameters studied (temperatures, pH, fat contents, protein contents, and rates of contamination with different germs) and to identify risk factors linked to this quality.

## 2. Material and Methods

### 2.1. Material

#### 2.1.1. Areas and Study Population

Our study identified five areas of the mid-northern region of Algeria (Algiers, Blida, Bouira, Boumerdes, and Tizi Ouzou). It included three dairies and five collection trucks, each equipped with an insulated tank and a pumping system. The selection criteria are based primarily on their location in the study region, as well as their acceptance to cooperate in this work.

#### 2.1.2. Survey of the Situation

A survey was performed to collect information about the farms, the various collectors, and dairies in order to characterize each link in the chain of milk production.

### 2.2. Methods

#### 2.2.1. Sampling Protocol

The study was conducted during the agricultural year which began on October 1, 2012, and ended September 30, 2013. Several tests were used at monthly intervals to assess the overall quality of raw milk tanks. Two periods were selected for this study: a cold period (P1: from early October to late March) and another hot one (P2: April 1 until the end of September). A total of 120 samples were collected and analyzed at two levels in the sector, (i) 60 from the collector's tank at the end of the collection (M1) and (ii) 60 taken from the same tanks at delivery (M2). All samples correspond to the morning milking. Two monthly samples were taken from each tank on the same day at M1 and M2. About 100 mL was taken aseptically in sterile test tubes from each tank for microbiological analysis. On the other hand, the same volume was collected for performing physicochemical analysis. All samples were stored at temperatures between 4 and 8°C in a cold box and transported to be analyzed immediately after arrival to the laboratory.

*(1) Physicochemical Analysis*. At the end of the collection of the milk and at the dairy processing units, the temperature and pH at 20°C were measured, respectively, by using a thermometer and a pH meter (Hanna Instruments, Italy). The fat content was determined by acidobutyrometric method of Gerber and the protein content was determined by the Kjeldahl method applied for milks.

*(2) Microbiological Analysis*. Subsequent decimal dilutions were prepared to 10^−7^. The counting was carried using a counting of colonies (Colony Counter SC6). All culture mediums were provided by the Pasteur Institute (Algeria).

Total mesophilic aerobic bacteria (TMAB) was enumerated on plate count agar (PCA) and incubated at 30°C for 72 hours. Coliforms bacteria were performed on citrate lactose agar with deoxycholate (DLC) 1‰. Plates were incubated for 24–48 hours at 30°C for TC and 44°C for FC. Yeasts and molds were enumerated on Sabouraud glucose medium at 4% and incubated for 5 days at about 25°C, where* Staphylococcus aureus* were determined on Baird Parker agar supplemented with egg yolk and potassium tellurite. For* Salmonella* spp. determination, a preenrichment of 25 mL of milk was carried out on broth of lactose mannitol buffered (BLMB) supplemented with additive, followed by an enrichment on sodium selenite broth. Counting and isolation were performed on Hektoen agar after 24 hours of incubation at 37°C. The method described by Harrigan and MacCance [12] was used for the determination of sulphite-reducing Clostridia.* Listeria monocytogenes *were determined by a prior enrichment in 25 mL of Fraser 1/2 broth, inoculation in Fraser broth, and isolation on Palcam agar.

*(3) Other Tests*. Reduction test of methylene blue (RTMB) was done using the method of Bonfoh et al. [13] and the detection of antibiotic residues using Delvotest SP-NT (DSM, Netherlands), [14].

#### 2.2.2. Statistical Analysis

The results of microbiological analyses were transformed into Log10 cfu/mL to achieve parametric statistical tests. Data were analyzed using ANOVA with the following modules of STATISTICA 8.0. The significance level was fixed at *P* < 0.05.

## 3. Results

### 3.1. Characteristics of Production Sites and Collector's Tank

A total of 21 farms covering 450 dairy cows were hit by our study with an average of 4 farms and 90 cows per tank (Table 1). Milking was done manually in only 2 farms. The milk tank was present in approximately 67% of farms. Otherwise, the milk was stored in a bucket which is cooled. In this category, the farmers hold no more than 10 dairy cows. The milk was transported to the dairy in a truck over an average distance of 27 km.

A sample of raw milk should be analyzed for the amount of fat it contains because it is the only criterion for compensation for these producers. Milk in dairies must undergo a heat treatment (pasteurization) or be fermented before being marketed.

### 3.2. Characteristics of Milks Collected at the Farm and at Delivery

#### 3.2.1. Physical and Nutritional Characteristics

Temperatures measured immediately at the end of collection (M1) were between 6°C and 15°C, with a mean value of 10,34 ± 0,75. At delivery (M2), a reduction of initial temperatures was observed with an average of 8,8 ± 0,4 (Table 2). Indeed, significant variations (*P* < 0.001) were observed for this parameter on departure and arrival of the tanks. The average pH decreased from 6,63 ± 0.027 in M1 to 6,62 ± 0,03 in M2. Indeed, 30% of samples (*n* = 18) had pH < 6,6 before leaving the farm, with 12 of them recorded in a hot period (P2). pH > 6,8 characterized 12% of milks analyzed, 86% of them in a cold period (P1). While, at M2, 32% of samples had pH < 6,6, 13 milks of them were found in P2. However, 8,33% of milks had pH > 6,8 all in P1.

Slight decreases were noted in the rate of fat (35,44 ± 1,87 against 35,01 ± 1,95) and protein rate (29,55 ± 0,44 against 29,2 ± 0,42), respectively, in M1 and M2. The analysis showed no significant variation (*P* > 0.05), during the same period in almost all physical and nutritional parameters studied (pH, fat, and protein contents).

#### 3.2.2. Microbiological Characteristics

The average contaminations in total mesophilic aerobic bacteria (TMAB), total coliforms (TC), feacal coliforms (FC), yeasts, and molds of the samples taken at the end of the collection (M1), were, respectively, 6,42 ± 0,43, 4,6 ± 0,41, 3,29 ± 0,47, 4,58 ± 0,29, and 3,23 ± 0,33. Just at arrival to the dairies at M2, these averages have evolved considerably to reach respective values for these germs of 7,5 ± 0,54, 5,31 ± 0,46, 4,29 ± 0,23, 5,34 ± 0,29, and 3,88 ± 0,24 (Table 3). These values were also higher in a hot period (P2) than in a cold period (P1).

Considering these two periods, the respective values of germs studied were 6,24 ± 0,14 and 6,6 ± 0,25 against 7,3 ± 0,13 and 7,69 ± 0,13 for TMAB, 4,18 ± 0,18 and 5,03 ± 0,39 against 4,83 ± 0,20 and 5,79 ± 0,44 for TC, and 2,63 ± 0,56, 3,95 ± 0,21 and 3,86 ± 0,28, 4,78 ± 0,20 for FC. Regarding yeasts and molds, average loads were, respectively, 4,57 ± 0,27 and 4,58 ± 0,19 against 5,26 ± 0,16 and 5,42 ± 0,22 in M2 (2,87 ± 0,33, 3,57 ± 0,50 in M1 and 3,62 ± 0,19, 4,14 ± 0,39 in M2). In addition, FC and molds which were absent at the beginning at P1 in, respectively, 8% and 5% of milks, were developed during transport to reach all samples arrived at different dairies. The change was described as follows: *P* < 0.001 during 2 periods for total mesophilic aerobic bacteria (TMAB), total coliforms (TC), feacal coliforms (FC), and yeasts and for molds at P1. The analysis also revealed positive correlations between TMAB and temperatures (*P* > 0.05), between TMAB and TC (*P* < 0.05), and for FC (*P* < 0.01).

Results obtained from the detection of pathogenic bacteria are presented in Table 4. It was shown in M1 that 20 cases of samples tested were positive for* S. aureus* and 42% for Clostridia. This contamination reached respectively 14 other and the half of milks in M2.

*Listeria monocytogenes* was detected in 25% of samples at M1, with an average count from 0 to 11 germs/25 mL. In M2, two other samples were achieved by a maximum of 32 germs. T5 was the most affected, during P1 for* S. aureus* and* Listeria* and during P2 for SRC. The results of analysis for the detection of* Salmonella *spp. showed no contamination in all samples analyzed. Antibiotic residues were detected in 35% of samples at delivery (M2).

#### 3.2.3. Microbial Contamination and Transport

The average reduction time of methylene blue (RTMB) experienced strong fall estimated at about 54% from M1 to M2. Significant changes were noted in M1 (*P* < 0.001) and in M2 (*P* < 0.05). The RTMB was positively correlated with pH, *P* < 0.01 at M1 and *P* > 0.05 at M2.

## 4. Discussion

The results showed a decrease in the average temperature between the end of collection (M1) and at delivery (M2) estimated at 1,54°C. These values are lower than those found by Gran et al. [15] and Bonfoh et al. [16]. The lowest temperatures at delivery were probably explained by the failure of the equipment of storage and cooling of milk in some farms. pH < 6,6 is related to the acidification of milk during transportation to the dairy unit, while pH > 6,8 observed in cold season was probably linked to the wetting of milk with water during this period in order to increase the income of the farmer through higher volumes of milk delivered. The weak decreases in the rates of fat and protein are considered to be the result of the presence of some psychrotrophic bacteria altering milk by their lipolytic and proteolytic properties [17]. These are mostly Pseudomonas:* Pseudomonas fluorescens*,* Pseudomonas fragi, *and* Alteromonas putrefaciens*. The results also clearly indicate that the average germ count in the samples studied at delivery was significantly higher compared to that of milk at the end of collection (M1). These milks were also higher in a hot season (P2) than in a cold season (P1). Coliforms bacteria loads were also significantly higher in summer than in winter in the study of Lues et al. [18]. Bouzaid et al. [19] indicate that average values on raw milks taken from the point of sale are superior to ours obtained in M2. The detection of coliforms and pathogenic flora in milks probably originated from cow's udder, milking utensils, or water used [20]. The presence of fecal coliforms indicates the possibility of fecal contamination and involves a risk that other enteric pathogens may be present in the milk [15, 21], while* Staphylococcus aureus* are particularly indicators of the presence of the subclinical mastitis in the dairy cattle [22]. They are dangerous because of their ability to potentially transmit from animals to humans and vice versa [23]. However, a good cooling reduces the number of Gram negative bacteria such as coliforms bacteria but has a little effect on Gram positive bacteria such as* Staphylococcus aureus* [24, 25]. Handling of milk could be one of the factors causing high loads in yeasts and molds in our study. These microorganisms were very often transferred from animals' feed to milk [26, 27]. The utensils used for milk collection at the farm are usually the largest source of contamination of milk [28–31]. Microbial contamination in raw milk depends not only on its contamination during milking and storage conditions but also on the temperature at which it was stored and the time that elapses between milking and collection [32]. The mode of delivery associated with a collection system observed in this present work allows the mixing of milks from different dairy farms. It is sufficient that the milk of one of them is not well preserved so that all the milk in the same tank is damaged. The number of dairy cows milked per tank was negatively correlated (*P* < 0.05) with the loads of TMAB and coliforms bacteria. However, the distance to the dairy unit and average counts in different germs were determined to be associated (*P* < 0.05). These observations are in agreement with the results of Gran et al. [15]. The level of microorganisms in milk is likely to increase with increasing time of delivery [33]. The presence of antibiotics in samples was linked to milk produced by cows in receipt of antimicrobial therapy. It also appears that the highest averages in all germs studied found in the T5 are considered to be a result, on the one hand, of the practice of manual milking (*P* < 0.05) and, on the other hand, of the buckets used (*P* < 0.05) for preserving milk in, respectively, 50% and 75% of farms associated with this tank. Traditional practices of milking identified have also been described by Lues et al. [18] and Belli et al. [34] to be likely to contribute to fecal contamination of milk and the proliferation of several microorganisms. T1 and T2 have also high rates of germs. This is probably related to the proportions of farmers who keep their milk in buckets cooled (33.33% for T2 and 40% for T1). However, the lowest germs count was observed in the remaining tanks. Note that the mechanical milking and the milk cooling equipment were widespread in all farms collected by these tanks.

## 5. Conclusion

The results of this study lead to the conclusion that the initial rate of contamination and season had significant effects on nutritional and hygienic quality of raw milk in this region. Other risk factors have also been identified in this study, namely: the practice of hand milking, absence of equipment for storing and cooling of raw milk at farm. Adding to all this the distance between the place of production and the place of delivery. Therefore, the implementation of a device on the standard milk hygiene is necessary and must involve all parties in the chain of milk production. This is through the improvement in terms of farm management, while supporting the breeder for advice on good husbandry practices. Every collector must detect a level of contamination by rapid tests to prevent the mixing of milks with different hygienic qualities. Another solution is to encourage the installation of collection centers near farms which do not have equipment for storage and refrigeration and are away from the dairy unit. The introduction of a payment system is based on the hygienic quality of milk to encourage breeders to improve their production. Finally, dairies must control milk at the reception in order to trace the producer to the origin of the defect in the tank.

## Figures and Tables

**Table 1:** Main characteristics of farms and collectors studied.

Characteristics	T1	T2	T3	T4	T5	Total/mean
Area of collection	Blida	Boumerdes	Alger	Tizi Ouzou	Bouira	5 areas

Number of farms/tank	5	6	2	4	4	21 **Mean = 4**

Average number of cows/tank	124	75	111	98	42	450 **Mean = 90**

Type of milking						
(i) Manual	0/5	0/6	0/2	0/4	2/4 **(50%)**	2/21 **(10%)**
(ii) Automatic	5/5 **(100%)**	6/6 **(100%)**	2/2 **(100%)**	4/4 **(100%)**	2/4 **(50%)**	19/21 **(90%)**

Number of farms						
(i) Milk tank	3/5 **(60%)**	4/6 **(66,7%)**	2/2 **(100%)**	4/4 **(100%)**	1/4 **(25%)**	14/21 **(66,7%)**
(ii) Cooled bucket	2/5 **(40%)**	2/6 **(33,3%)**	0/2 **(0%)**	0/4 **(0%)**	3/4 **(75%)**	7/21 **(33,3%)**

Average distance of farms/dairy (km)	15,5	7	14	16	82,5	135 **Mean = 27**

T: tank.

**Table 2:** Results of physicochemical analysis of samples at different levels of the production chain.

Parameters	M1			M2		
	Minimum	Mean ± SD	Maximum	Minimum	Mean ± SD	Maximum
*T* (°C)	6	10,34 ± 0,75	15	6	8,8 ± 0,41	13
pH	6,46	6,63 ± 0,02	6,91	6,45	6,62 ± 0,03	6,9
Fat (g/kg)	30	35,44 ± 1,87	41	30	35,01 ± 1,95	41
Protein (g/kg)	27	29,55 ± 0,44	33	26	29,2 ± 0,42	32

SD: standard deviation. M1: at the end of collection (at farm). M2: at delivery (at the dairy unit).

**Table 3:** Microbiological characteristics of milks analyzed (expressed in log⁡10 cfu/mL).

	TMAB			TC			FC			Yeasts			Molds		
	Min	Mean ± SD	Max	Min	Mean ± SD	Max	Min	Mean ± SD	Max	Min	Mean ± SD	Max	Min	Mean ± SD	Max
M1	5,21	6,42 ± 0,43	8,32	2,51	4,6 ± 0,41	6,06	0	3,29 ± 0,47	5,07	3,12	4,58 ± 0,29	5,71	0	3,23 ± 0,33	4,82
M2	6,27	7,5 ± 0,54	9,15	3,77	5,31 ± 0,46	7,46	2,8	4,29 ± 0,23	6,08	3,83	5,34 ± 0,29	6,25	2,04	3,88 ± 0,24	5,31
P1M1	5,21	6,24 ± 0,14	8,32	2,51	4,18 ± 0,18	5,22	0	2,63 ± 0,56	4,26	3,12	4,57 ± 0,27	5,41	0	2,87 ± 0,33	4,82
P1M2	6,43	7,3 ± 0,13	8,83	3,9	4,83 ± 0,20	5,86	2,8	3,86 ± 0,28	4,96	3,83	5,26 ± 0,16	6,1	2,04	3,62 ± 0,19	5,31
P2M1	5,47	6,6 ± 0,25	8,3	3,47	5,03 ± 0,39	6,06	2,76	3,95 ± 0,21	5,07	3,51	4,58 ± 0,19	5,71	0	3,57 ± 0,50	4,8
P2M2	6,27	7,69 ± 0,13	9,15	3,77	5,79 ± 0,44	7,46	3,6	4,78 ± 0,20	6,08	4,53	5,42 ± 0,22	6,25	2,04	4,14 ± 0,39	4,98

M1: at the end of collection (at farm). M2: at delivery (at the dairy unit). P1: cold period. P2: hot period.

**Table 4:** Frequency of pathogens and antibiotic residues in the milks studied.

Samples	*Staphylococcus aureus *	Sulfite-reducing Clostridia	*Listeria monocytogenes *	Antibiotic residues
	Number (%)	Number (%)	Number (%)	Number (%)
P1M1	9/30 **(30%)**	12/30 **(40%)**	8/30 **(26,66%)**	—
P1M2	15/30 **(50%)**	17/30 **(56,66%)**	9/30 **(30%)**	9/30 **(30%)**
P2M1	11/30 **(36,66)**	13/30 **(43,33%)**	7/30 **(23,33%)**	—
P2M2	19/30 **(63,33%)**	13/30 **(43,33%)**	8/30 **(26,66%)**	12/30 **(40%)**
M1	20/60** (33,33%)**	25/60** (41,66%)**	15/60** (25%)**	—
M2	34/60** (56,66%)**	30/60** (50%)**	17/60 ** (28,33%)**	21/60 **(35%)**
